# STAMP: Spatial-Temporal Anchored Motion Planning for Zero-Shot Continuous Vision-and-Language Navigation

**DOI:** 10.3390/s26123698

**Published:** 2026-06-10

**Authors:** Tai Liu, Xiaoyan Qi, Liuyi Wang, Jinlong Li, Xiao Lin, Minghao Zhu, Yulong Cui, Chengju Liu, Qijun Chen

**Affiliations:** 1 CRRC Qingdao Sifang Co., Ltd., No. 88 Jinhong East Road, Chengyang District, Qingdao 266111, China; sf-liutai@cqsf.com (T.L.); cuiyulong@cqsf.com (Y.C.); 2 College of Electronics and Information Engineering, Tongji University, No. 4800 Cao’an Road, Jiading District, Shanghai 201804, China; 2331853@tongji.edu.cn (X.Q.); li_jinlong@tongji.edu.cn (J.L.); 2111118@tongji.edu.cn (X.L.); zmhh_h@tongji.edu.cn (M.Z.); liuchengju@tongji.edu.cn (C.L.); qjchen@tongji.edu.cn (Q.C.)

**Keywords:** vision-and-language navigation, embodied AI, visual navigation

## Abstract

Vision-and-Language Navigation in continuous environments (VLN-CE) requires embodied agents to ground natural language instructions into reliable long-horizon motion decisions under partial observability. Despite their strong semantic understanding and reasoning abilities, Multimodal Large Language Model (LVLM) struggle when directly applied to VLN, as they lack explicit spatial grounding, embodied memory, and awareness of geometric and reachability constraints, leading to perceptual misalignment and cascading decision errors in complex scenes. To address these limitations, we propose STAMP, a Spatial-Temporal Anchored Motion Planning framework for zero-shot VLN-CE, which systematically bridges the gap between pretrained world knowledge and embodied navigation. STAMP adopts a hierarchical design that decouples high-level semantic reasoning from low-level motion execution, enabling a frozen LVLM to operate over a structured, navigation-oriented abstraction. Its core novelty lies in a multimodal spatial-temporal anchoring mechanism that explicitly encodes instruction-relevant landmarks, action semantics, depth-aware geometry, and historical navigation context, together with an explicit Chain-of-Navigation reasoning process that constrains decision-making to navigation-critical cues. Furthermore, STAMP incrementally constructs an online, backtracking-enabled topological map, supporting robust planning under uncertainty. Extensive experiments demonstrate the effectiveness of the proposed STAMP framework, achieving performance comparable to state-of-the-art zero-shot methods on VLN-CE benchmarks and in real-world settings.

## 1. Introduction

Vision-and-Language Navigation in Continuous Environments (VLN-CE) [1,2,3,4,5] requires an embodied agent to execute natural language instructions through continuous motion in unfamiliar 3D environments, relying solely on raw egocentric observations and low-level control. Unlike classical SLAM [6,7], which requires metric mapping and explicit goal coordinates, our approach enables an agent to navigate directly from free-form natural language instructions without any pre-built map. Unlike idealized settings with predefined viewpoints or connectivity [8,9,10,11,12], VLN-CE demands online perception, spatial reasoning, and action selection under partial observability. This task introduces several challenges, including complex instruction parsing, vision-language understanding, awareness of spatial constraints, and persistent embodied memory to support exploration and backtracking, and recovery from suboptimal decisions. Existing VLN-CE approaches [13,14,15,16] largely rely on in-domain expert demonstrations and task-specific training, which could introduce dataset bias that limits sim-to-real generalization, thereby hindering reliable zero-shot deployment on physical robots in real-world environments.

The advent of Large Vision-and-Language Models (LVLMs) [17,18,19] offers a promising paradigm for alleviating these limitations. Pretrained on massive image-text corpora, LVLMs encode rich world knowledge and commonsense reasoning capabilities, endowing them with the potential to generalize to unseen scenes, interpret ambiguous instructions, and operate under real-world variability, which are the key prerequisites for deployable VLN systems. Nevertheless, directly applying LVLMs to VLN-CE remains nontrivial, and existing LVLM-based VLN methods exhibit several critical shortcomings. First, existing LVLM-based approaches feed fragmented sub-images (e.g., candidate-viewpoint crops or equirectangular panoramic patches) as separate visual inputs [20,21,22,23,24], which disrupts holistic panoramic understanding and degrades geometric consistency. Second, the lack of explicit semantic emphasis and structured guidance across visual, linguistic, and memory modalities often causes diffused attention and hallucinated reasoning during inference [22,25]. Third, task-specific models [13,26,27] require extensive dataset-specific training and do not generalize zero-shot, while LVLM-based methods that forgo structured memory [21,28] struggle with long-range backtracking when navigation errors occur.

**Figure 1:**
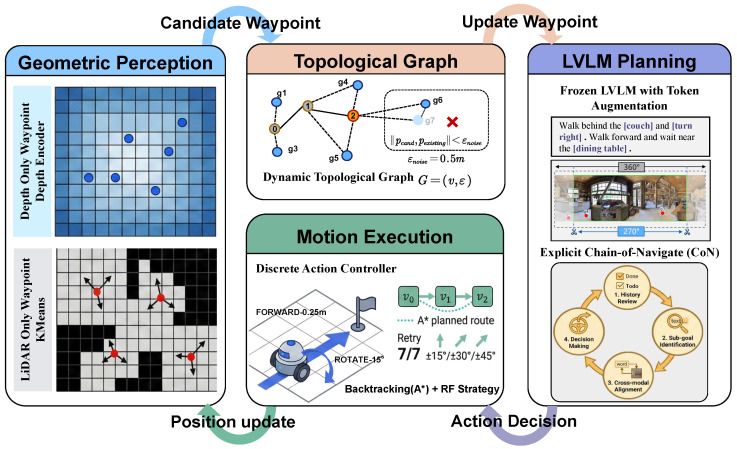
Overview of the proposed STAMP VLN-CE framework. The agent takes panoramic visual observations, depth maps, and language instructions as input to produce high-level navigation decisions, which are subsequently translated into low-level actions to reach the target location.

To address the aforementioned challenges, we propose STAMP (**S**patial-**T**emporal **A**nchored **M**otion **P**lanning), a hierarchical zero-shot navigation framework for VLN-CE, as illustrated in Figure 1. STAMP is designed to systematically bridge the gap between pretrained multimodal world knowledge and embodied navigation by introducing explicit spatial-temporal grounding, structured reasoning, and global memory, enabling fully deployment without task-specific fine-tuning.

To explicitly strengthen semantic guidance during navigation, we introduce a novel Multimodal Anchored Marking (MAM) mechanism that enriches semantic cues from three complementary perspectives: visual observation, language instruction, and historical memory. For *visual perception*, STAMP adopts continuous 360° panoramic imagery as the primary input, eliminating viewpoint fragmentation and providing a coherent global spatial context. Candidate waypoints are explicitly overlaid onto the panoramic view as visually anchored markers, enabling the LVLM to directly perceive their spatial locations and relative geometry within continuous space. For *language instructions*, we extract and emphasize landmark and action-related tokens using structured symbolic annotations, guiding the LVLM to focus on navigation-critical semantic elements rather than diffuse textual details. For *historical memory*, the historical navigation steps are encoded using temporally ordered action arrows and timestamps, which are persistently embedded into the panoramic image sequence. This design preserves spatial-temporal continuity across steps and allows past decisions to remain explicitly visible to the model. This tri-modal anchoring strategy converts abstract navigation reasoning into concrete spatial-temporal references across modalities. These references are easier for LVLMs to interpret and help activate their latent spatial association and reasoning capabilities.

Building upon this representation, we further design an explicit Chain-of-Navigation (CoN) reasoning template to enhance robustness in long-horizon decision-making. CoN structures the LVLM reasoning process through iterative task-progress review, subgoal decomposition, and cross-modal alignment. This design guides attention toward navigation-relevant cues and reduces spurious correlations and hallucinated reasoning. Additionally, this structured reasoning process also improves the interpretability and transparency of navigation decisions, enabling clearer analysis of model behavior in complex environments. Furthermore, to support reliable planning under uncertainty, we propose to construct an online Vision-and-Language Topological Map (VLTM) that serves as a persistent anchored memory. The map encodes visited locations, semantic observations, and navigational connectivity. It is explicitly incorporated into the LVLM prompt to support backtracking and long-range replanning when the agent detects a potentially suboptimal decision.

From a system perspective, STAMP decouples high-level semantic planning from low-level motion execution to ensure robustness and efficiency. The frozen LVLM is exclusively responsible for discrete waypoint selection based on the augmented multimodal prompt. Geometric feasibility and motion execution are handled by a lightweight, depth-dominant waypoint predictor and a discrete action controller. On the VLN-CE benchmarks [1,29], STAMP substantially outperforming existing zero-shot baselines. More importantly, we validate STAMP on a custom-built service robot in real-world environments, achieving an 88% zero-shot SR across 25 diverse tasks and demonstrating its practical effectiveness and deployability.

Our contributions are summarized as follows:

- We propose STAMP, a novel Spatial-Temporal Anchored Motion Planning framework for zero-shot VLN-CE that effectively bridges pretrained multimodal world knowledge with embodied navigation.
- We introduce a Multimodal Anchored Marking (MAM) mechanism that explicitly enhances navigation-relevant semantics across visual observations, language instructions, and historical memory, providing structured spatial-temporal grounding for LVLM-based decision-making.
- We develop an online Vision-and-Language Topological Map (VLTM) with backtracking-aware prompting, enabling robust planning under uncertainty and persistent embodied memory for long-term navigation.
- We present an explicit Chain-of-Navigation (CoN) reasoning template that organizes long-horizon navigation through task-progress retrospection, subgoal decomposition, and cross-modal alignment, improving both planning robustness and decision interpretability.

The remainder of this paper is organized as follows. Section 2 surveys related work on VLN-CE and zero-shot VLN approaches leveraging LLMs and LVLMs. Section 3 formally defines the VLN-CE task formulation. Section 4 presents the proposed STAMP framework in detail. Section 5 reports comprehensive experimental results on standard benchmarks and real-world deployment. Section 6 discusses the limitations of this study and outlines directions for future work. Finally, Section 7 concludes the paper with insights and promising future directions.

## 2. Related Work

### 2.1. Vision-and-Language Navigation

Navigation is a core capability of embodied intelligence [30,31,32], yet achieving reliable performance in open and unstructured real-world environments remains challenging due to partial observability, cross-modal fusion, and long-horizon reasoning. Vision-and-Language Navigation (VLN) [8] advances this objective by enabling embodied agents to follow natural language instructions grounded in visual perception, typically within predefined connectivity graphs. However, such idealized abstractions limit practical deployment. Therefore, Vision-and-Language Navigation in Continuous Environments (VLN-CE) was introduced [1]. Built upon the Habitat simulator [33], VLN-CE allows agents to navigate freely in continuous 3D space using low-level actuation, thereby more faithfully reflecting real-world navigation complexity and challenges.

Over the past several years, the VLN-CE community has witnessed remarkable progress, including the development of cross-modal fusion [34,35,36,37,38,39,40,41], historical memory encoding [42,43], data augmentation [3,10,16,44], and reinforcement learning [1,4,45], and more recently, large foundation models [26,46,47,48,49,50,51]. From an architectural perspective, VLN-CE approaches can be categorized into two paradigms: *end-to-end* and *hierarchical* decision-making. End-to-end methods directly fuse visual, linguistic, and historical memory features into a unified policy network, typically outputting dense low-level actions (e.g., move forward 0.25 m, turn left/right 15°) or sequences of dense waypoints [52,53,54]. In contrast, hierarchical frameworks decouple navigation into two stages [2,13,55]: a waypoint prediction module that generates candidate subgoals from the surrounding environment, and a waypoint selection module that integrates multi-modal inputs to choose the next navigational target.

In the early development of VLN-CE, most approaches relied on task-specific compact models trained via supervised or reinforcement learning. While these methods achieved competitive performance on in-domain benchmarks, they suffered from significant performance degradation when deployed in out-of-distribution environments due to domain shift and limited generalization. With the recent breakthrough of large language models (LLMs) [19,56] and large vision language models (LVLMs) [17,57,58,59], a promising avenue has emerged: leveraging their zero-shot reasoning capabilities to tackle VLN-CE without task-specific fine-tuning. Accordingly, this work primarily focuses on harnessing the zero-shot potential of LVLMs to address the VLN-CE challenge.

### 2.2. Zero-Shot VLN with Large Foundation Models

The advent of LLMs and LVLMs has enabled zero-shot VLN, where agents can follow instructions and navigate in unknown environments without task-specific fine-tuning. Existing methods typically exploit foundation models for spatial grounding, memory construction, and navigation planning. Some methods integrate LLMs with explicit spatial representations, such as occupancy maps, vision-language maps, or topological maps, where LLMs parse instructions and structured maps ground target locations or support path planning [23,60,61,62,63,64]. Other methods enhance visual grounding and waypoint selection through LVLM prompting. For example, CoNVOI [65] annotated numbered regions within the visual frame for path construction, AO-Planner [25] introduces affordance-oriented prompting based on SAM-segmented navigable regions, and InstructNav [20] builds multi-source value maps to support zero-shot planning.

Beyond spatial grounding, recent methods incorporate navigation history, future exploration, and collaborative reasoning to improve long-horizon decision-making. NavGPT [22] uses visual observations, navigation history, and future explorable directions for LLM-based decision-making, while SmartWay [24] improves waypoint prediction with history-aware reasoning. OpenNav [29] explores open-source LLMs for zero-shot VLN-CE by combining different LLMs for collaborative perception and reasoning. CA-Nav [66] further formulates zero-shot VLN-CE as a sequential, constraint-aware sub-instruction completion process.

Despite these advances, existing zero-shot VLN-CE methods face persistent challenges. Visual observations are often spatially limited and temporally fragmented. This deficiency frequently leads to navigation failures, particularly in long-horizon tasks requiring sustained reasoning over extended trajectories. To address these limitations, we propose STAMP, a zero-shot VLN-CE framework that explicitly organizes multi-step reasoning through a multimodal anchored token augmentation mechanism and an online vision-and-language topological map, enabling reliable planning and execution for VLN-CE.

## 3. Task Formulation

VLN-CE [1] is an embodied AI task that requires an agent to navigate freely in continuous 3D space by executing low-level motor primitives, guided solely by natural language instructions and egocentric visual observations. The agent operates in a continuous 3D environment E represented by photorealistic indoor scenes. At time step *t*, the agent’s state is fully characterized by its pose:

(1) $$s_{t}=\left(x_{t},y_{t},z_{t},\theta_{t},\phi_{t}\right)\in S,$$

where $\left(x_{t},y_{t},z_{t}\right)\in R^{3}$ denotes the global Cartesian coordinates, and $\left(\theta_{t},\phi_{t}\right)\in \left[0,2\pi \right)$ represent the yaw and pitch angles, respectively. t each time step, the agent receives a multi-modal observation $o_{t}=\left(V_{t},D_{t},I\right)\in O$, where $V_{t}\in R^{12\times H\times W\times 3}$ denotes a panoramic egocentric RGB observation composed of 12 uniformly discretized views at 30° intervals; $D_{t}\in R^{12\times H\times W\times 1}$ represents the corresponding depth maps (optional, and currently only used for waypoint generation in simulation); and $I=\{w_{1},w_{2},\ldots ,w_{N}\}$ is a natural language instruction consisting of *N* tokens that describe the navigation goal.

In the habitat simulator [33], the agent selects discrete low-level actions from A={move_forward,turn_left,turn_right,stop}, where each action induces a deterministic state transition governed by the environment dynamics $s_{t+1}=f\left(s_{t},a_{t}\right)$. By default, move_forward advances the agent by a fixed distance $\delta_{\mathrm{move}}=0.25$ m in its current heading direction, while rotation actions adjust the yaw angle by $\delta_{\theta }=\pm 15{}^{\circ}$. The agent’s behavior is defined by a policy $\pi :\{H_{t},O_{t},I\}\to A$, which maps the observation history $H_{t}=\left\{o_{0},a_{0},\ldots ,o_{t-1},a_{t-1},o_{t}\right\}$ to an action. The resulting trajectory is a sequence $\tau =\{s_{0},a_{0},s_{1},a_{1},\ldots ,s_{T},a_{T}\}$, where *T* is the episode length terminated by the stop action or a maximum step limit. An episode is deemed successful if the geodesic distance from the agent’s final position to the target location is within a threshold:

(2) $$\mathrm{Success}=I\left[d_{\mathrm{geo}}\left(s_{T},s_{\mathrm{goal}}\right)\leq \epsilon_{\mathrm{tol}}\right],$$

where $d_{\mathrm{geo}}\left(\;\cdot \;,\;\cdot \;\right)$ denotes the shortest collision-free path distance, I(·) is the indicator function, and $\epsilon_{\mathrm{tol}}=3$ m is the commonly adopted tolerance threshold.

## 4. Method

This section presents STAMP, our spatial-temporal anchored motion planning framework for zero-shot VLN-CE. We first describe the waypoint prediction mechanism for both simulated and real-world settings (Section 4.1). To support long-horizon navigation, we construct an online Vision-and-Language Topological Map (VLTM) that maintains persistent multimodal history (Section 4.2). Next, we introduce the Multimodal Anchored Marking (MAM) mechanism for augmenting LVLM inputs with structured spatial-temporal and semantic cues (Section 4.3). We then present an explicit Chain-of-Navigation (CoN) reasoning template that guides the LVLM in selecting the next navigation action (Section 4.4). Following each high-level decision, the agent executes the selected action via a low-level controller and updates its state accordingly (Section 4.5). This process iterates until the stop action is predicted or a predefined step limit is reached.

### 4.1. Depth-Driven Waypoint Prediction

Reliable waypoint prediction is a fundamental component of our navigation framework, as it defines the discrete action space over which the LVLM performs reasoning and selects the next navigable location. To this end, we propose a geometry-driven prediction paradigm that bridges the perception-action gap while avoiding overfitting to RGB semantics. The design is motivated by two key considerations.

First, traversability is inherently a geometric property. The inclusion of RGB features can introduce spurious correlations between navigable directions and domain-specific appearance patterns. Second, discretizing the continuous action space into a structured set of candidate waypoints provides a natural interface between frozen LVLMs and low-level control. While LVLMs are well suited for selecting among discrete, well-defined options, direct end-to-end visuomotor control remains unstable and difficult to generalize.

In simulation, the waypoint predictor follows the standard R2R-CE action granularity, with $\delta_{\mathrm{move}}=0.25$ m and $\delta_{\theta }=3{}^{\circ}$. In the real-world LiDAR pipeline, the local traversability grid spans a 3 m sensing radius at 0.05 m/cell resolution; free-space extraction applies a 3×3 morphological erosion (1 iteration) to remove isolated cells at obstacle boundaries. Candidate waypoints are obtained by running Faiss K-means on the navigable point set with $k=\min(N_{\mathrm{wp}},\;\lfloor |P|/10\rfloor )$ clusters and 20 iterations, where $N_{\mathrm{wp}}=5$ is the maximum desired waypoint count and |P| is the number of navigable cells. Each cluster center is then verified within a 0.5 m safety radius; unsafe centers are iteratively displaced away from the nearest obstacle until the safety constraint is satisfied or discarded after 10 failed attempts. Finally, waypoints closer than 1.0 m to each other are pruned to ensure spatial diversity. These parameters were kept fixed across all reported experiments.

**Depth-driven prediction (simulation).** Given a panoramic depth map $D_{t}\in R^{12\times H\times W\times 1}$ at time step *t*, we first extract hierarchical spatial features through a frozen ResNet-50 backbone which is pre-trained on an end-to-end pointGoal Navigation task [67]:

(3) $$F_{t}=\mathrm{ResNet}50\left(D_{t}\right).$$

Freezing the backbone avoids catastrophic forgetting and retains the geometric feature representations acquired during large-scale pretraining. The flattened spatial features are concatenated with a sinusoidal directional encoding that conditions the network on the agent’s current heading $\left(\theta_{t},\phi_{t}\right)$:

(4) $$e_{t}^{\mathrm{dir}}={\left[\sin\theta_{t},\;\cos\theta_{t},\;\sin\phi_{t},\;\cos\phi_{t}\right]}^{⊤},$$

(5) $$z_{t}=\left[\mathrm{Flatten}\left(F_{t}\right);\;e_{t}^{\mathrm{dir}}\right].$$

The fused representation $z_{t}$ is decoded by an *L*-layer Transformer decoder to generate a traversability heatmap. A set of *k* learnable positional query embeddings $h^{\left(0\right)}\in R^{k\times d_{\mathrm{model}}}$ serves as the decoder input. At each layer l∈{1,…,L}, the queries attend over the encoded context $z_{t}$ via scaled dot-product multi-head cross-attention:

(6) $$Q^{\left(l\right)}=h^{\left(l-1\right)}W_{Q}^{\left(l\right)},\;K^{\left(l\right)}=z_{t}W_{K}^{\left(l\right)},\;V^{\left(l\right)}=z_{t}W_{V}^{\left(l\right)},$$

(7) $${\mathrm{Attn}}^{\left(l\right)}=\mathrm{softmax}\;\left(\frac{Q^{\left(l\right)}{\left(K^{\left(l\right)}\right)}^{\;⊤}}{\sqrt{d_{k}}}\right)V^{\left(l\right)},$$

where $W_{Q}^{\left(l\right)},W_{K}^{\left(l\right)},W_{V}^{\left(l\right)}\in R^{d_{\mathrm{model}}\times d_{k}}$ are learnable projection matrices and $d_{k}=d_{\mathrm{model}}/n_{\mathrm{head}}$ is the per-head dimension. The attention output is residually connected, layer-normalized, and passed through a two-layer feed-forward network (FFN):

(8) $$h^{\left(l\right)}=\mathrm{LayerNorm}\;\left({\mathrm{Attn}}^{\left(l\right)}+\mathrm{FFN}\;\left({\mathrm{Attn}}^{\left(l\right)}\right)\right),$$

(9) $$\mathrm{FFN}\left(x\right)=W_{2}\;\mathrm{ReLU}\;\left(W_{1}x+b_{1}\right)+b_{2}.$$

The final decoder output $h^{\left(L\right)}$ is first projected through a linear layer followed by a ReLU activation σ(·) to obtain an intermediate representation:

(10) $$B_{t}=\sigma \;\left(W_{\mathrm{out}1}\;h^{\left(L\right)}+b_{\mathrm{out}1}\right).$$

This intermediate feature is then passed through a second linear head to produce the traversability heatmap:

(11) $$H_{t}=W_{\mathrm{out}2}\;B_{t}+b_{\mathrm{out}2},$$

where each cell value represents the predicted probability of navigability in the corresponding spatial region. Candidate waypoints are extracted by applying Non-Maximum Suppression (NMS) to $H_{t}$, retaining k=5 local maxima with an angular suppression radius $r_{\mathrm{nms}}=15{}^{\circ}$ to ensure spatial diversity:

(12) $$W_{t}^{\mathrm{sim}}=\mathrm{NMS}\;\left(H_{t},\;k=5,\;r_{\mathrm{nms}}\right).$$

Each selected pixel location $\left(u_{i},v_{i}\right)$ is back-projected into 3D agent-centric coordinates by reading the corresponding depth value $d_{i}=D_{t}\left[u_{i},v_{i}\right]$ and applying the pinhole camera model:

(13) $$p_{i}=d_{i}\;\cdot \;K^{-1}{\left[u_{i},\;v_{i},\;1\right]}^{⊤},$$

where K is the camera intrinsic matrix. This yields a set of candidate 3D positions ${\left\{p_{i}\right\}}_{i=1}^{k}$ defining the agent’s local navigable waypoints at step *t*.

**LiDAR geometric clustering (real-world).** In physical deployment, panoramic RGB-D cameras are often less accessible and entail higher hardware costs, in addition to being susceptible to specular reflections, overexposure. We therefore adopt a LiDAR-based geometric pipeline for real-world waypoint prediction. Given the raw point cloud $P_{t}={\left\{p_{j}\right\}}_{j=1}^{M}$ with $p_{j}\in R^{3}$, we first project all returns onto a top-down 2D occupancy grid $G_{t}\in {\left\{0,1\right\}}^{W_{g}\times H_{g}}$ at resolution *r*:

(14) $$G_{t}\left[u,v\right]=I\;\left[\exists \;p_{j}\in P_{t}:u=\;\left\lfloor p_{j}^{x}/r\right\rfloor ,\;v=\;\left\lfloor p_{j}^{y}/r\right\rfloor ,\;p_{j}^{z}\in \left[z_{\min},z_{\max}\right]\right],$$

where the height filter $\left[z_{\min},z_{\max}\right]$ retains only points within the navigable vertical band, discarding ground reflections and overhead structures. To build in a conservative safety margin for the robot’s physical footprint, the occupied binary map undergoes morphological erosion with a disk-shaped structuring element B of radius $r_{e}$:

(15) $${\tilde{G}}_{t}=G_{t}⊖B_{r_{e}},$$

where ⊖ denotes the erosion operator. The free traversable cells within a bounded planning horizon $d_{\max}$ from the agent’s grid coordinate $\left(u_{0},v_{0}\right)$ are collected as:

(16) $$F_{t}=\left\{\left(u,v\right)\;|\;{\tilde{G}}_{t}\left[u,v\right]=0,\;{∥\left(u,v\right)-\left(u_{0},v_{0}\right)∥}_{2}\leq d_{\max}/r\right\}.$$

K-Means clustering partitions $F_{t}$ into *N* spatial groups with initial centroids ${\left\{\mu_{n}^{\left(0\right)}\right\}}_{n=1}^{N}$, providing diverse spatial coverage of the free space. The raw cluster centers are subsequently refined by iterative repulsive potential field optimization, which pushes each centroid away from nearby obstacle cells $O_{n}=\{o|{\tilde{G}}_{t}\left[o\right]=1,\;∥o-\mu_{n}{∥}_{2}\leq r_{\mathrm{rep}}\}$:

(17) $$\mu_{n}^{\left(l+1\right)}=\mu_{n}^{\left(l\right)}+\eta \sum_{o\in O_{n}}\frac{\mu_{n}^{\left(l\right)}-o}{∥\mu_{n}^{\left(l\right)}{-o∥}_{2}^{2}+\epsilon },$$

where η is the step size and ϵ is a small stabilization constant that prevents division by zero when a centroid coincides with an obstacle boundary. This iterative refinement guarantees that the converged waypoints $W_{t}^{\mathrm{real}}={\left\{\mu_{n}^{*}\right\}}_{n=1}^{N}$ maintain adequate clearance from all obstacle surfaces. Notably, the entire LiDAR pipeline is free of learnable parameters, as each operation, including projection, erosion, and clustering is formulated as a closed-form geometric computation. This design enables the waypoint prediction stage to transfer seamlessly to novel environments without requiring domain adaptation or retraining.

### 4.2. Online Vision-and-Language Topological Map

**Figure 2:**
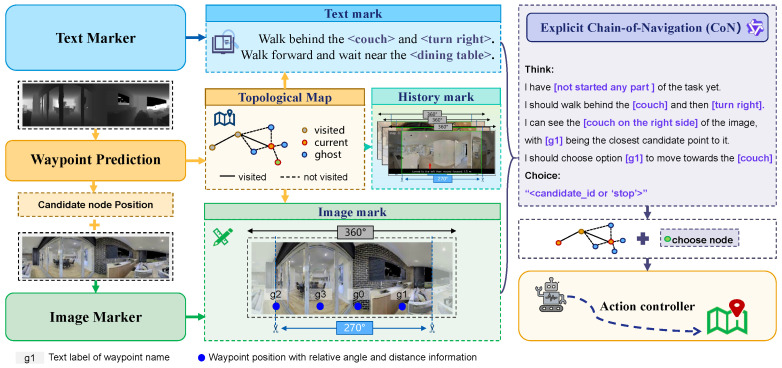
Overview of the proposed STAMP framework. It incorporates three types of markers: text, image, and history markers. For each modality, the corresponding marker is injected into the input stream to highlight critical guidance cues. An online topological map is employed to maintain navigation history, and an explicit chain-of-navigation is introduced to guide the LVLM in reasoning through the navigation process in a more structured and reliable manner.

Building upon the predicted waypoints, we construct and incrementally maintain a dynamic topological graph G=(V,E), where nodes represent navigable positions and edges encode their connectivity relationships. As illustrated in Figure 2, the node set V consists of two types: *visited nodes*, corresponding to positions physically reached by the agent and indexed as integers (0, 1, 2, …), and *ghost nodes*, which denote predicted but unvisited candidate locations (prefixed with “g”, e.g., “g1”, “g2”). For each node, we store the associated panoramic observation along with its relative spatial relationship to the current position, forming a vision-and-language topological map.

To prevent node redundancy caused by revisiting similar spatial regions, we implement a spatial deduplication mechanism: for each candidate waypoint $w_{c}$, we compute its Euclidean distance to all existing nodes $v_{j}\in V$ as $\mathrm{dist}\left(w_{c},v_{j}\right)={∥p_{w_{c}}-p_{v_{j}}∥}_{2}$. If $\min_{j}\mathrm{dist}\left(w_{c},v_{j}\right)<\tau_{\mathrm{overlap}}$ (with $\tau_{\mathrm{overlap}}=0.5$ m accounting for localization noise), $w_{c}$ is merged with the nearest existing node; otherwise, a new ghost node is created. Edge weights in E represent geodesic distances between connected nodes, computed through Dijkstra’s algorithm on the evolving graph structure.

At each step, the agent is provided with the candidate waypoints connected to its current node and selects one unvisited node to proceed. When no unvisited forward waypoints are available, the agent instead receives a summary of all unvisited nodes in the topological map, including their coordinates relative to the current position, their associated visual appearances, and the step index at which each was first observed. The agent then chooses either to stop or to select one of these nodes as a backtracking target. Therefore, this topological representation provides a compact yet expressive spatial abstraction that supports both forward exploration and backward retracing to previously observed locations.

### 4.3. Multimodal Anchored Marking (MAM)

To bridge the modality gap between frozen-parameter LVLMs and embodied navigation tasks, we introduce a structured multimodal token augmentation mechanism that transforms abstract spatial reasoning into concrete visual-language references. This mechanism operates across three complementary modalities—textual instructions, visual observations, and historical trajectories—without requiring model fine-tuning. Each augmentation strategy explicitly encodes navigation-critical information as symbolic tokens embedded within the original input stream, effectively guiding the LVLM’s attention toward geometrically feasible actions while suppressing irrelevant contextual noise.

#### 4.3.1. Textual Instruction Augmentation

Natural language navigation instructions inherently follow an action-landmark sequential structure (e.g., “turn left at the kitchen, then proceed toward the dining table”). However, verbose human expressions often dilute critical semantic units with redundant discourse markers and adverbial modifiers, causing attention dispersion in long-sequence processing. To address this, we apply explicit lexical marking to navigation-critical tokens using angle brackets 〈·〉, yielding structured prompts such as “turn 〈left〉 at the 〈kitchen〉”. This augmentation serves dual purposes: first, it functions as a soft attention prior that biases the LVLM’s cross-attention weights toward spatially salient entities; second, it disambiguates homonymous references (e.g., distinguishing “left” as a directional verb versus a positional adjective) through syntactic isolation.

To enable the large model to accurately emphasize landmarks and actions in long navigation instructions, we design the following in-context prompting strategy:


**Instruction Augmentation Prompt**
Mark key {object_type} in long navigation instructions using {marker_type}.
**Examples:**
*Input:* Turn right at stairs and go down them. Stop between the bathroom and laundry rooms.*Output:* <Turn right> at <stairs> and <go down> them. <Stop between> the <bathroom> and [laundry rooms].*Input:* Turn completely around. Walk straight ahead through the doorway that is next to the giant urn.*Output:* <Turn completely around>. <Walk straight> ahead through the <doorway> that is next to the giant <urn>.

In addition to instruction augmentation, we design a dedicated system prompt for the VLN task. By explicitly defining the agent’s role, task, and input semantics, the prompt guides the LVLM to produce appropriate navigation decisions:


**System Prompt for VLN**

**Role:**
You are an indoor mobile robot tasked with completing a human-specified navigation instruction. Use the provided history video and current candidate point images to plan your next action efficiently. Key landmarks and actions in the instruction are marked with {mark_type}.
**Input Details:**
**History Video:** Displays the previously traversed trajectory and decisions (e.g., step 1 → step 2 → step 3). Use this information to track progress and avoid revisiting explored areas.**Current Observation:** A 360° panoramic image.–**Robot Front View (270° Horizontal Field of View):** The green rectangle indicates the central 270-degree horizontal field of view, covering 135° to both the left and right of the current heading. Objects within this region correspond to the robot’s forward-facing view, while objects outside this region lie behind or at extreme side angles.–**Vertical Axis (Depth Cue):** Objects appearing closer to the bottom of the image are physically nearer to the robot, regardless of their horizontal position.**Candidate Points:** Discrete navigable locations (e.g., g21, g23) that the robot can move to next. Some candidates may correspond to previously visited locations and should be avoided unless recovery from an error is required.


#### 4.3.2. Visual Observation Augmentation

**Figure 3:**
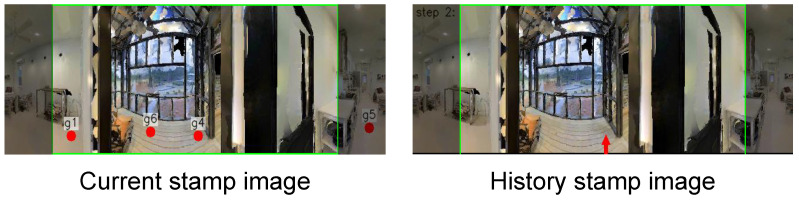
The current STAMP image augmentation includes gemometric waypoints, black semi-transparent rear masking, and green frontal highlighting. The history STAMP augmentation further incorporates red arrows and step counters.

Raw panoramic inputs present two fundamental challenges for LVLMs in navigation: (1) the absence of explicit action affordances, as pixels alone do not indicate traversable directions; (2) the 360° field of view introduces substantial visual redundancy from previously traversed rearward regions. As shown in Figure 3, our augmentation strategy addresses both issues through geometric token injection.

Specifically, candidate waypoints predicted by the depth-driven module (Section 4.1) are projected onto the panoramic RGB image as colored circular markers with alphanumeric labels (e.g., “g1”, “g2”). Given a waypoint in the local 3D coordinate frame p=(x,y,z), we first compute its azimuth and elevation angles:

(18) $$\theta =\mathrm{atan}2\left(x,z\right),\;\phi =\arcsin\;\left(\frac{y}{∥p∥}\right).$$

For an equirectangular panoramic image of width *W* and height *H*, the corresponding 2D image coordinates (u,v) are obtained via:

(19) $$u=\frac{\theta +\pi }{2\pi }\;W,\;v=\left(1-\frac{\phi +\frac{\pi }{2}}{\pi }\right)H.$$

To further constrain the LVLM’s spatial reasoning scope, we apply Gaussian masking with σ=30° to rear-view sectors beyond ±135° relative to the agent’s forward direction, encouraging forward-oriented decision making.

#### 4.3.3. Historical Trajectory Augmentation

Long-horizon navigation requires maintaining spatiotemporal consistency across decision steps, yet standard LVLMs lack explicit mechanisms for trajectory memory. Instead of encoding history as verbose textual summaries (which suffer from information loss and token inefficiency), we preserve the visual-action coupling of past states through chronologically arranged image and text sequences. The most recent N=12 steps of navigation history are offered as a horizontal strip of token-augmented panoramic frames positioned above the current observation.

Crucially, each historical frame retains its original waypoint markers and action-direction arrows, creating a visual trajectory log that encodes both spatial progression and decision rationale. To prevent context window overflow during extended navigation, we implement a dynamic forgetting mechanism: when step count exceeds $N_{\max}=12$, frames beyond this threshold are compressed into a short textual summary while preserving the most recent *N* visual frames. This hybrid representation balances memory fidelity with computational efficiency. Specifically, each compressed step is encoded as a tuple of relative action direction (one of five categories: *ahead* (±25° of the forward direction), *left* (25°–135° to the left), *right* (25°–135° to the right), *behind* (beyond 135° from forward), or *back to previous step*) and the corresponding travel distance. A representative summary takes the form: *“Step 1. Turned to the right then moved forward 1.8 m. Step 2. Turned to the left then moved forward 1.0 m. …”* This compact textual encoding preserves the essential trajectory structure while substantially reducing token consumption.

Together, these three augmentation strategies convert raw multimodal inputs into structured navigation-specific representations. By injecting geometric priors and temporal continuity cues as visual-language tokens, they compensate for the LVLM’s limited embodied experience while preserving its semantic reasoning ability, enabling zero-shot navigation without architectural modification or task-specific fine-tuning.

### 4.4. Explicit Chain-of-Navigation (CoN)

To further enhance decision rationality and interpretability, we design an explicit Chain-of-Navigation (CoN) reasoning template that guides the LVLM through four structured reasoning steps before outputting an action decision: (1) *Progress recap*: summarize completed subtasks based on historical trajectory; (2) *Subgoal extraction*: identify the immediate atomic objective (e.g., “turn toward corridor”); (3) *Cross-modal alignment*: match the subgoal’s semantic concepts with visual entities or spatial directions in current observation; (4) *Action decision*: select the geometrically and semantically consistent next waypoint. This constrained reasoning process significantly reduces hallucination and repetitive behaviors compared to unconstrained chain-of-thought prompting, while maintaining computational efficiency by limiting unnecessary verbose reasoning. The LVLM outputs a discrete action choice among candidate nodes (e.g., “g1”, “g2”) or termination commands (“stop”, “return”), which is then passed to the low-level controller.

### 4.5. Discrete Action Controller

The discrete action controller translates high-level waypoint selections into executable atomic actions A={FORWARD(0.25m),ROTATE_LEFT/RIGHT(15°),STOP}. When the target node is a direct neighbor of the current position in the topological graph, the controller employs a rotate-then-forward (RF) strategy: it first computes the relative azimuth $\theta_{\mathrm{rel}}$ and Euclidean distance $d_{\mathrm{rel}}$ between current and target positions, then executes $\left\lceil \theta_{\mathrm{rel}}/15{}^{\circ}\right\rceil$ rotation actions followed by $\left\lceil d_{\mathrm{rel}}/0.25\;m\right\rceil$ forward actions. To handle motion deadlocks caused by obstacles (detected when two consecutive FORWARD actions yield zero displacement), we implement a “tryout” recovery mechanism that attempts lateral rotations following a predefined sequence (±15°,±30°,±45°) with intermittent forward attempts until successful displacement occurs or a maximum retry count (e.g., 7 attempts) is reached. For non-adjacent target nodes (e.g., historical nodes requiring backtracking or distant ghost nodes), the controller first performs global path planning via Dijkstra algorithm on the topological graph to obtain an intermediate node sequence $\left\{v_{0}\to v_{1}\to \cdots \to v_{n}\right\}$. This hierarchical control design ensures robust execution while maintaining strict decoupling between semantic reasoning and motion control.

## 5. Experiments

We evaluate STAMP across three dimensions: (1) ablation studies of multimodal token augmentation; (2) comparison with recent zero-shot and supervised methods; (3) real-world deployment on a custom service robot. Simulation experiments use Habitat 0.2.1 with Matterport3D (MP3D) scenes; real-world tests span five indoor environments with diverse layouts and lighting conditions.

### 5.1. Experimental Setup

**Simulation Environment.** We adopt the Room-to-Room in Continuous Environments (R2R-CE) benchmark [1] built upon MP3D’s 90 photorealistic indoor scenes [68]. Following standard protocol, we evaluate on *val_seen* for ablation studies and *val_unseen* for cross-scene generalization. The agent receives 360° panoramic RGB observations (12×3 sub-images stitched to 224×768 resolution) and depth maps at each step. Action space consists of discrete primitives: FORWARD (0.25 m), ROTATE_LEFT/RIGHT (15°), and STOP. Maximum episode length is capped at 500 steps.

**Figure 4:**
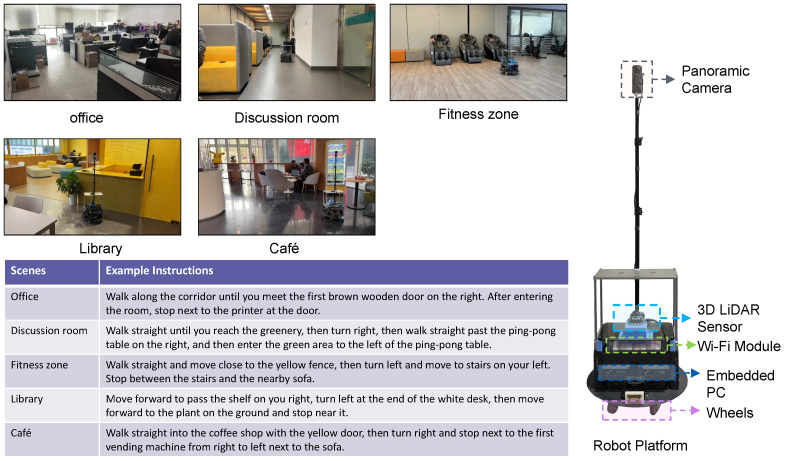
Custom service robot platform and five representative indoor scenes used for real-world deployment. The platform comprises four main components: (1) Insta360 X4 panoramic camera; (2) Livox Mid-360 LiDAR; (3) Orin Nano compute module; and (4) omnidirectional mobile base. A representative natural-language instruction is provided for each scene.

**Real-World Platform.** As shown in Figure 4, we deploy STAMP on a custom wheeled service robot equipped with an Insta360 X4 panoramic camera, Livox Mid-360 LiDAR, and NVIDIA Jetson Orin Nano. The robot operates in five representative indoor scenes: office (cluttered workspaces), discussion room (low-light furniture-dense area), fitness zone (glass reflections), library (long corridors), and café (dynamic pedestrians). Each scene contains 5 diverse navigation instructions with trajectory lengths from 3 m to 10 m, totaling 25 test episodes. No prior maps are loaded. SLAM (Cartographer) builds occupancy grids online.

**Evaluation Metrics.** We report four standard VLN metrics. (1) Success Rate (SR), defined as the percentage of episodes in which the agent stops within 3 m of the target while keeping the target in view. (2) Oracle Success Rate (OSR), defined as the percentage of episodes in which the agent reaches within 3 m of the target at any point along the trajectory. (3) Navigation Error (NE), defined as the final distance to the target for failed episodes. (4) Success weighted by Path Length (SPL), which measures the trade-off between success rate and path efficiency through their harmonic mean.

### 5.2. Ablation Studies and Single-Step Decision Analysis

Table 1 presents the ablation results on the R2R-CE *val_seen* split, evaluating the contributions of augmented markers across three modalities: image, history, and text.

**Image Modality Augmentation.** Explicit waypoint marking contributes most significantly, as its removal leads to a 10.8% drop in SR (from 35.2% to 24.4%). This result confirms that geometric action affordances need to be explicitly injected for frozen LVLMs to effectively reason about navigable directions. In addition, rear-view masking provides modest improvements, increasing SR by 1.9%, by reducing distractions from previously traversed areas. We further analyze the effect of the number of candidate waypoints presented to the waypoint predictor, as shown in Table 2. With only 1 or 2 candidates, the agent severely underperforms (SR = 2.0% and 20.0%, respectively), as insufficient candidates limit spatial exploration. Performance peaks at 5 waypoints (SR = 39.0%, SPL = 28.8%), which provides a balanced trade-off between action diversity and prompt clarity. Increasing the number further to 8 or 12 degrades SR, suggesting that an excess of candidates introduces decision ambiguity that the frozen LVLM struggles to resolve.

**History Modality Augmentation.** Image-sequence history outperforms video or text alternatives by 6.0% and 8.4% and in SR, respectively. Removing history entirely causes SR to plummet to 20.4%, validating long-horizon dependency in VLN-CE. Directional arrows contribute most (+8.7% SR), resolving orientation ambiguity in ego-centric sequences. Additionally, we analyze the effect of history length on navigation performance. As shown in Table 3, a history length of 12 achieves the best overall performance, with the lowest NE (7.27 m) and the highest SR (39%). Increasing the history length to 15 yields only a marginal SPL gain but reduces SR, suggesting that overly long histories introduce redundant context and impair decision-making. In Table 4, we further compare template-based and LLM-based summarization for actions beyond the memory window. The template-based approach achieves better performance with lower computational cost. Removing explicit historical step indices or action offsets from the template also degrades performance, further validating the effectiveness of our design.

**Text Modality Augmentation.** Removing the angle-bracket markup 〈·〉 from both action and landmark tokens causes SR to drop from 35.2% to 30.6% and SPL from 28.1% to 24.0%. Ablating landmark marking and action marking individually degrades SPL by 2.7% and 0.9%, respectively, confirming that explicit textual annotation of both token types contributes to navigation efficiency.

**Figure 5:**
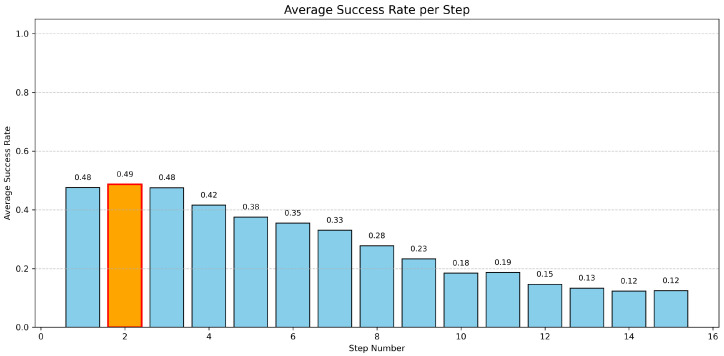
Single-step decision accuracy across trajectory lengths for STAMP. Orange highlighting indicates the statistical result with the highest average success rate.

Crucially, Figure 5 reveals that STAMP achieves 48.7% single-step decision accuracy–nearly half of all steps select the optimal waypoint toward the goal at the first 3 steps. This finding is pivotal: despite suboptimal decisions at individual steps, the hierarchical architecture with topological backtracking enables error correction across the trajectory. Specifically, when the agent deviates from the optimal path (e.g., due to ambiguous visual cues), the dynamic graph structure allows revisiting historical nodes or exploring alternative “ghost nodes,” transforming a sequence of 50% accurate decisions into a 35.2% end-to-end success rate. This error-resilient property distinguishes STAMP from end-to-end approaches where single-step errors propagate irreversibly.

**Figure 6:**
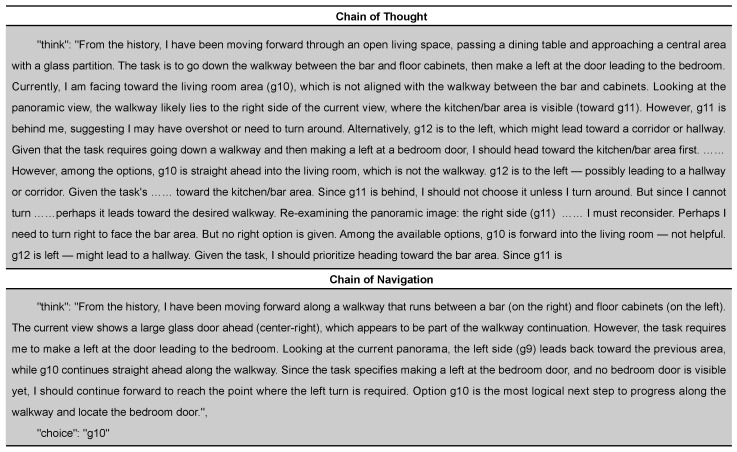
CoN vs. CoT reasoning on trajectory #1219, step 4. CoN anchors reasoning to spatial layout (“walkway between bar and cabinets”) and defers turning until the bedroom door is visible; CoT fixates on linguistic parsing and proposes an invalid left turn despite geometric inconsistency.

**Chain-of-Navigation Reasoning.** Explicit CoN reasoning improves SR by 2.4% over unconstrained generation in multi-turn corridors. As shown in Figure 6, CoN produces concise, geometry-grounded reasoning (e.g., “continue straight along walkway to locate bedroom door”) versus CoT’s verbose chains prone to direction confusion.

### 5.3. Base Model Comparison and Scaling Analysis

Table 5 compares navigation performance across LVLM architectures and scales on 100 episodes from R2R-CE *val_unseen*. Three critical findings emerge:

First, parameter scale exhibits diminishing returns beyond 8B. Qwen3-VL-2B (16.0% SR) and Qwen3-VL-4B (15.0% SR) show minimal gains, while the jump to 8 B yields a modest improvement (16.0% SR). The most significant leap occurs at 32 B (39.0% SR), after which scaling to 235 B yields only +3.0% SR. This saturation effect indicates that 8–32 B represents the optimal cost-performance frontier for embodied navigation. Larger models primarily benefit from improved world knowledge and enhanced spatial reasoning.

Second, architectural improvements outweigh pure scale. Qwen3-VL-32B (39.0% SR) substantially outperforms Qwen2.5-VL-72B (29.0% SR) despite having fewer parameters, demonstrating that Qwen3’s enhanced visual encoder and MRoPE position encoding provide more effective spatial grounding than parameter inflation. The generational gap is most pronounced at equivalent scales: Qwen3-VL-32B achieves nearly double the SR of Qwen2.5-VL-32B (20.0% SR).

Third, well-designed prompting allows open-source models to achieve competitive performance against closed-source APIs. With OpenNav, Qwen3-VL-2B obtains a 16.0% SR, approaching GPT-4V’s 19.0% SR despite its much smaller scale, while Qwen3-VL-32B surpasses Open-Nav by 20.0% absolute SR. These results suggest that structured multimodal prompting can partly offset model-scale limitations and offers a practical route toward deployable and auditable navigation systems without relying on external APIs.

### 5.4. Comparison with State-of-the-Art Methods

Table 6 compares STAMP against representative methods on R2R-CE *val_unseen*. STAMP achieves higher SR (32.8%) and SPL (25.25%) among other zero-shot methods using a frozen open-sourced Qwen3-VL-32B backbone. Against open-source zero-shot baselines, STAMP outperforms SmartWay [24] by +3.8% SR, CA-Nav [66] by +7.5%, A^2^Nav [69] by +10.2%, and MapGPT (CE) [23] by +12.7%. It also surpasses InstructNav [20] (+1.8% SR) and OpenNav [29] (+13.8% SR), which rely on closed-source LVLM APIs. Compared to the supervised method, ETPNav [13] (57.0% SR), STAMP narrows the gap to 24.2% without any task-specific training, demonstrating its strong cross-scene generalization and deployment practicality.

### 5.5. Real-World Deployment Validation

**Figure 7:**
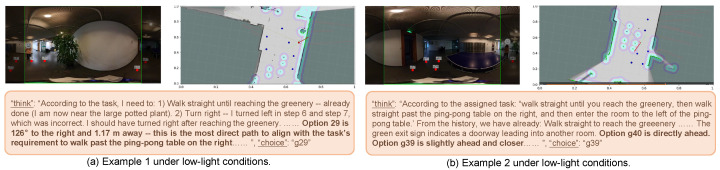
Qualitative examples of the agent’s real-world navigation under low-light conditions.

We deploy STAMP on the physical robot platform across 25 trajectories in five indoor scenes. As summarized in Table 7, the system achieves 88% SR and 96% OSR without any fine-tuning on real data. This experimental result validates strong sim-to-real transferability. Office scenes yield the highest success rate due to clear landmarks (e.g., “printer”, “penguin cabinet”) and structured layouts. As shown in Figure 7, our method shows good generalization in low-light scenarios, such as the discussion room. This benefits from the LVLM’s strong visual perception and natural-language instruction understanding capabilities.

**Figure 8:**
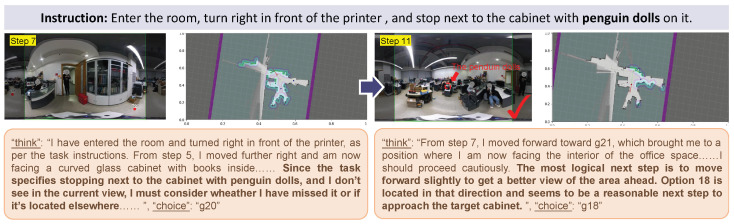
Qualitative analysis of the agent’s navigation in the real-world office scenario. At step 7, the agent cannot find the required penguin doll and believes it has missed it. Therefore, it backtracks to the previous node g18 at step 11, where it successfully finds the penguin doll.

As shown in Figure 8, in the office scenario, the agent successfully navigates to the “cabinet with penguin dolls” despite an initial misalignment. When the direct path is obstructed, the agent leverages topological backtracking to revisit a previously unvisited historical node (g18), which was predicted at step 5, demonstrating robust exploration under occlusion. This result validates the effectiveness of the proposed topological map construction and backtracking mechanism.

## 6. Discussion

STAMP demonstrates that frozen LVLMs, when equipped with structured multimodal prompting, can achieve competitive zero-shot navigation without task-specific training. The 48.7% single-step accuracy, when combined with topological error correction, yields 32.78% end-to-end success. This experimental result validates our hypothesis that hierarchical decomposition mitigates LVLM’s spatial reasoning limitations. Model scaling analysis reveals that architectural improvements (Qwen3 vs. Qwen2.5) provide greater gains than pure parameter inflation, with 8–32B representing the optimal deployment frontier. Real-world results confirm practical sim-to-real transferability, though challenges remain in glass-rich environments and scenarios prone to odometry drift, which are well-known limitations of LiDAR-based online mapping and represent inherent constraints of the current system design.

**Figure 9:**
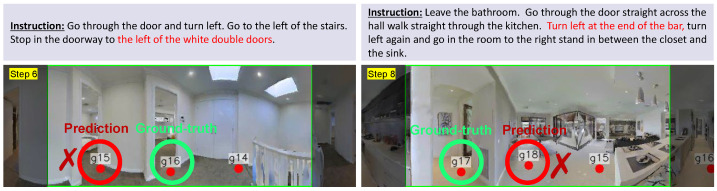
Qualitative analysis for some failure cases on the R2R-CE val-unseen set.

Figure 9 presents qualitative analyses of representative failure cases. We observe that the LVLM may incorrectly infer explicit directional cues at certain steps, reflecting a common limitation of current LVLMs in embodied directional perception. Recent studies, such as [70], have explored this issue and may provide useful directions for future improvement.

For the real-world evaluation, each of the 5 indoor scenes is paired with 5 natural-language instructions, yielding 25 trajectories in total. On average, each instruction comprises 26.12 words, 5.28 action clauses, and 3.64 landmark references, reflecting a moderate level of linguistic complexity. We acknowledge that this evaluation is constrained in scale due to the limited availability of diverse real-world test environments. It is therefore intended as a practical deployment validation rather than a statistically exhaustive benchmark. A larger and more rigorously controlled real-world study is planned for future work. We also note that the reliance on a 360° panoramic camera imposes additional hardware requirements that may limit deployment on platforms equipped only with monocular sensors. Extending the framework to monocular or multi-camera rigs is a direction for future work.

## 7. Conclusions

We present STAMP, a zero-shot hierarchical navigation framework that bridges the modality gap between frozen-parameter multimodal large language models and embodied navigation through structured multimodal token augmentation. By explicitly injecting geometric action affordances via lexical marking, visual waypoint overlay, and chronologically arranged image-sequence history, STAMP enables robust zero-shot planning without task-specific fine-tuning. Extensive evaluation on R2R-CE benchmarks and real-world deployment across five indoor scenes validates its strong sim-to-real transferability and generalization to novel landmarks. Current limitations include inference latency and waypoint prediction degradation in glass-rich environments. Future work will address these via edge-accelerated inference and multi-sensor fusion, while extending toward long-horizon embodied reasoning with language-conditioned object manipulation.

## Figures and Tables

**Table 1:** Ablation study on R2R-CE *val_seen*. Best results in **bold**.

Config	NE (m) ↓	OSR (%) ↑	SR (%) ↑	SPL (%) ↑
Full STAMP	**6.6**	**47.3**	**35.2**	**28.1**
Image				
no wp	7.6	33.4	24.4	20.3
no rear	7.0	44.6	33.3	25.5
no front	7.0	45.5	31.9	24.8
History				
no arrow	7.7	44.3	26.5	18.5
no step	7.9	37.5	24.3	18.7
Img hist	6.8	45.0	33.6	26.6
Vid hist	7.7	43.4	27.6	20.8
Txt hist	7.8	38.7	25.2	19.4
None	8.1	40.4	20.4	14.0
Text				
no 〈·〉	7.1	43.8	30.6	24.0
Ldmk only	6.8	44.3	32.3	25.4
Act only	6.7	46.1	35.0	27.2
Reasoning				
no CoN	6.8	46.0	32.8	25.3

**Table 2:** Waypoint number analysis on Open-Nav val-unseen set.

Waypoint Num.	NE (m) ↓	OSR (↑)	SR (↑)	SPL (↑)
1	8.71	2.0	2.0	2.0
2	8.33	25.0	20.0	15.8
3	7.80	38.0	32.0	23.6
**5**	7.27	46.0	**39.0**	**28.8**
8	**6.30**	**50.0**	38.0	27.6
12	7.48	38.0	29.0	21.5

**Table 3:** History length analysis on Open-Nav val-unseen set with different history frame lengths.

History Length	NE (m)	OSR (%) ↑	SR(%) ↑	SPL (%) ↑
1	7.59	38.0	23.0	16.0
3	7.88	**47.0**	32.0	21.7
6	7.74	45.0	35.0	24.8
9	7.97	43.0	36.0	25.3
**12**	**7.27**	46.0	**39.0**	28.8
15	7.61	43.0	37.0	**29.1**

**Table 4:** Comparison of history summarization methods.

Method	NE (m) ↓	OSR (%) ↑	SR(%) ↑	SPL(%) ↑
LLM	7.55	45.0	35.0	26.7
Template	**7.27**	**46.0**	**39.0**	**28.8**
- w/o Step	7.67	44.0	32.0	23.4
- w/o Offset	7.88	45.0	34.0	25.2

**Table 5:** Performance comparison of Qwen-VL models under local vLLM deployment on the Open-Nav val-unseen set [29]. AET and AST are reported as mean ± standard deviation.

Model	Scale	AET ↓ (s)	AST ↓ (s)	OSR ↑ (%)	SR ↑ (%)	SPL ↑ (%)
Open-Nav	10 B	162.4 ± 9.0	27.0 ± 3.6	23.0	19.0	16.1
STAMP (Qwen2.5-VL)	7 B	42.5 ± 8.7	2.9 ± 0.3	37.0	15.0	8.2
	32 B	58.8 ± 7.9	4.0 ± 0.3	36.0	20.0	10.9
	72 B	57.7 ± 17.9	4.8 ± 0.3	42.0	29.0	19.7
STAMP (Qwen3-VL)	2 B	39.7 ± 5.4	2.6 ± 0.4	40.0	16.0	7.2
	4 B	41.6 ± 12.5	3.0 ± 0.4	33.0	15.0	7.8
	8 B	38.1 ± 18.8	3.5 ± 0.4	33.0	16.0	11.3
	32 B	54.9 ± 24.3	5.4 ± 1.1	**46.0**	**39.0**	**28.8**

**Table 6:** Comparison with previous methods on R2R-CE *val_unseen*. ^‡^: Closed-source LVLM API.

Method	Zero-Shot	NE (m) ↓	OSR (%) ↑	SR (%) ↑	SPL (%) ↑
Seq2Seq [1]	✗	7.37	40.0	32.0	30.00
CMA [1]	✗	6.20	52.0	41.0	36.00
ETPNav [13]	✗	4.76	63.7	57.0	49.03
InstructNav ^‡^ [20]	✓	6.89	-	31.0	24.00
SmartWay [24]	✓	7.01	**51.0**	29.0	22.46
OpenNav ^‡^ [29]	✓	6.70	23.0	19.0	16.00
MapGPT (CE) [23]	✓	8.56	32.8	20.1	14.83
A^2^Nav [69]	✓	-	-	22.6	11.10
CA-Nav [66]	✓	7.58	48.0	25.3	10.80
**STAMP (Ours)**	✓	6.84	46.0	**32.8**	**25.25**

**Table 7:** Real-world navigation performance across five indoor scenes (25 trajectories total).

Scene	Number of Traj.	SR (%) ↑	OSR (%) ↑
Office	5	100.0	100.0
Discussion room	5	80.0	100.0
Fitness zone	5	80.0	100.0
Library	5	80.0	100.0
Café	5	80.0	80.0
**Overall**	**25**	**88.0**	**96.0**

## Data Availability

The used VLN-CE dataset is available at https://github.com/jacobkrantz/VLN-CE (accessed on 27 May 2026). The data presented in this study are available on request from the corresponding authors.
